# Shared prenatal impacts among childhood asthma, allergic rhinitis and atopic dermatitis: a population-based study

**DOI:** 10.1186/s13223-019-0365-y

**Published:** 2019-09-03

**Authors:** Ching-Heng Lin, Jiun-Long Wang, Hsin-Hua Chen, Jeng-Yuan Hsu, Wen-Cheng Chao

**Affiliations:** 1 Department of Medical Research, Taichung Veterans General Hospital, 1650 Taiwan Boulevard, Sect. 4, Taichung, 40705 Taiwan; 2 Department of Healthcare Management, National Taipei University of Nursing and Health Sciences, Taipei, Taiwan; 3 Department of Public Health, College of Medicine, Fu Jen Catholic University, Taipei, Taiwan; 4 Division of Chest Medicine, Department of Internal Medicine, Taichung Veterans General Hospital, Taichung, Taiwan; 5 Department of Life Sciences, National Chung-Hsing University, Taichung, Taiwan; 6 Institute of Biomedical Science and Rong Hsing Research Center for Translational Medicine, Chung-Hsing University, Taichung, Taiwan; 7 Division of Allergy, Immunology and Rheumatology, Department of Internal Medicine, Taichung Veterans General Hospital, Taichung, Taiwan; 8 School of Medicine, National Yang-Ming University, Taipei, Taiwan; 9 Institute of Public Health and Community Medicine Research Center, National Yang-Ming University, Taipei, Taiwan; 10 Department of Industrial Engineering and Enterprise Information, Tunghai University, Taichung, Taiwan; 11 School of Physical Therapy, Chung-Shan Medical University, Taichung, Taiwan; 12 School of Medicine, China Medical University, Taichung, Taiwan; 13 Department of Nursing, College of Medicine & Nursing, Hung Kuang University, Taichung, Taiwan; 14 Department of Business Administration, National Changhua University of Education, Changhua, Taiwan

**Keywords:** Asthma, Allergic rhinitis, Atopic dermatitis, Prenatal factors, Perinatal factors, Birth cohort

## Abstract

**Background:**

Increasing prevalence of childhood allergic diseases including asthma is a global health concern, and we aimed to investigate prenatal risk factors for childhood asthma and to address the potential shared prenatal impacts among childhood asthma, allergic rhinitis (AR) and atopic dermatitis (AD).

**Methods:**

We used two claim databases, including Taiwan Birth Cohort Study (TBCS) and National Health Insurance Research Database (NHIRD), to identify independent paired mother–child data (mother–child dyads) between 2006 and 2009. The association between prenatal factors and asthma was determined by calculating adjusted odds ratio (aOR) with 95% confidence interval (CI) using conditional logistic regression analysis.

**Results:**

A total of 628,878 mother–child dyads were included, and 43,915 (6.98%) of children developed asthma prior to age 6. We found that male gender (aOR 1.50, 95% CI 1.47–1.53), maternal asthma (aOR 1.80, 95% CI 1.71–1.89), maternal AR (aOR 1.33, 95% CI 1.30–1.37), preterm birth (aOR 1.32, 95% CI 1.27–1.37), low birth weight (aOR 1.14, 95% CI 1.10–1.19) and cesarean section (aOR 1.10, 95% CI 1.08–1.13) were independent predictors for childhood asthma. A high urbanization level and a low number of older siblings were associated with asthma in a dose–response manner. Notably, we identified that the association between maternal asthma and childhood asthma (aOR 1.80, 95% CI 1.71–1.89) was stronger compared with those between maternal asthma and childhood AR (aOR 1.67, 95% CI 1.50–1.87) as well as childhood AD (aOR 1.31, 95% CI 1.22–1.40). Similarly, the association between maternal AR and childhood AR (aOR 1.62, 95% CI 1.53–1.72) was higher than those between maternal AR and childhood asthma (aOR 1.33, 95% CI 1.30–1.37) as well as childhood AD (aOR 1.35, 95% CI 1.31–1.40). Furthermore, the number of maternal allergic diseases was associated with the three childhood allergic diseases in a dose–response manner.

**Conclusions:**

In conclusion, this population-based study provided evidence of prenatal impacts on childhood asthma and demonstrated the shared maternal impacts among childhood asthma, AR, and AD. These findings highlight the shared prenatal impacts among allergic diseases, and studies are warranted to address the pivotal pathway in allergic diseases.

## Background

The prevalence of childhood allergic diseases, including asthma, allergic rhinitis (AR) and atopic dermatitis (AD), has increasingly risen worldwide in the past decades [1, 2]. A number of birth cohorts have found that prenatal factors, including maternal and perinatal factors, may lead to the development of childhood asthma, mainly maternal asthma, a high urbanization level, a low number of older siblings, preterm delivery, a low birth weight, and cesarean section [3–5]. The aforementioned three childhood allergic diseases have been described as an atopic march in childhood, indicating the shared immunologic feature of allergic responses [6, 7]. Recently, two large-scale genome-wide association studies (GWAS) have discovered a number of highly shared genetic susceptible loci among asthma, AR and AD; therefore, these three allergic diseases might have shared mother–child associations [8, 9]. However, there is limited evidence, particularly in population-based birth cohorts, to address the impact of prenatal factors on these three childhood allergic diseases. Herein, we used two population-based claim databases, including the birth cohort and national health insurance databases in Taiwan, to investigate the association between maternal as well as perinatal factors and incident childhood asthma and to further address the potential shared mother–child associations among childhood asthma, AR, and AD.

## Methods

### Ethical statements

This study was approved by the Institutional Review Board of Taichung Veterans General Hospital, Taiwan (IRB number CE17178A). Informed consent was not required given that the used claim databases in the present study contain only de-identified data.

### Data sources

The present study consists of two population-based claim databases, including the Taiwan Birth Cohort Study (TBCS) database and the National Health Insurance Research Database (NHIRD). TBCS was initiated in 2003 under the auspice of the Health Promotion Administration in Taiwan and has routinely collected perinatal data in Taiwan since 2003 [10]. Taiwan launched a single-payer National Health Insurance (NHI) program on March 1, 1995. As of 2015, 99.6% of Taiwan’s population was enrolled in the NHI program [11]. NHIRD contains registration files and original claim data for reimbursement and is maintained by the National Health Research Institutes (NHRI) for research purposes. In this study, we used perinatal data between 2006 and 2009 in TBCS and linked the perinatal data with NHIRD to obtain relevant medical information regarding allergic diseases of mothers and children.

### Definition of allergic diseases

The outcome of the present study was the development of childhood allergic diseases including asthma, AR, and AD. The diagnosis of the allergic diseases was in accordance with the International Classification of Diseases, 9th Revision, Clinical Modification (ICD-9-CM), and the ICD-9-CM codes for asthma, AR and AD were 493, 477 and 691, respectively. Subjects with the aforementioned allergic diseases were defined as having at least three ambulatory visits or one hospital admission with a diagnosis of the allergic disease. Importantly, to accurately define asthma, we restricted the diagnosis of asthma by the ever prescription of inhaled corticosteroid (ICS) given that ICS is the fundamental therapy in asthma [12]. Additionally, we also restricted the diagnosis of AR by the prescription of the nasal corticosteroid spray and AD by the persistence of AD after age 3 [13].

### Covariates

The perinatal data, including, maternal age at delivery (years), gestational age (weeks), parity, birth weight (g), birth length (cm), and model of delivery were obtained from TBCS, while the maternal allergic diseases were determined by the same ICD-9-CM code as mentioned. We also included gestational diabetes mellitus (ICD-9-CM codes, 648.0, 648.8 and 775.0) and preeclampsia (ICD-9-CM codes, 642.4, 642.5, 642.6, and 642.7) as covariates given that gestational diabetes mellitus and preeclampsia might potentially be associated with childhood allergic diseases [14, 15]. Urbanization levels were classified into four clusters based on population density (people/km^2^), population ratio of elder subjects aged over 65 years, population ratio of subjects with college or above educational levels, population ratio of agriculture workers, and the number of physicians per 100,000 subjects [16].

### Statistical analysis

Data were presented as the mean (standard deviation (SD)) for continuous variables and as number (percentages) for categorical variables. The differences were analyzed using the Student’s *t*-test for continuous variables and Pearson’s χ^2^ test for categorical variables. A conditional logistical regression model was conducted to estimate the adjusted odds ratio (aOR) and 95% confidence interval (CI) of incident childhood allergic diseases including asthma, AR and AD after adjustment for relevant covariates. All data were analyzed using statistical software version 9.3 (SAS Institute, Inc., Cary, NC, USA). A p-value < 0.05 was considered statistically significant.

## Results

### Demographic characteristics

A total of 791,746 infants were identified between 2006 and 2009 in the present study.

**Fig. 1:**
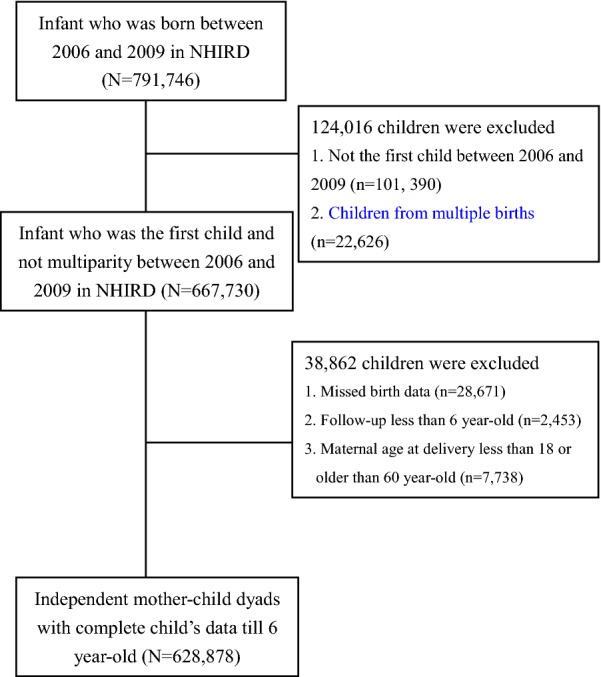
Flow chart of subjects’ enrollment

Given that one mother who had more than one child during the study period may lead to the correlated mother–child dyads and the inevitable correlated mother–child dyads in children of twins or other multiple births, we hence included the first child born during the study period and excluded children from multiple births. Therefore, all of the mother–child dyads in the present study were independent mother–child dyads. Children without complete birth data (n = 28,671), whose follow-up duration was less than 6 years (n = 2453), and whose maternal age at delivery was younger than 18 or older than 60 years (n = 7738) were also excluded. Then, a total of 628,878 independent mother–child dyads were eligible for analyses in this study (Fig. 1). Of these children with the complete medical record until age 6, 43,915 (6.98%) of them developed asthma. In addition to asthma, we also noted that AR (7808, 1.24%) and AD (26,901, 4.28%) were also prevalent childhood allergic diseases. With regards to maternal allergic diseases, the prevalence of maternal allergic diseases in asthma, AR and AD were 2.24%, 12.5% and 1.74%, respectively (Table 1). Taken together, these data showed that childhood allergic diseases were prevalent in the present study, with approximately 7% of children developed asthma prior to 6-year-old.

**Table 1:** Demographic data of enrolled subjects

Variable	N = 628,878 (100%)
Childhood allergic diseases	
Sex	
Female	300,290 (47.7)
Male	328,588 (52.3)
Allergic diseases	
Asthma	43,915 (6.98)
AR	7808 (1.24)
AD	26,901 (4.28)
Maternal allergic diseases and perinatal variables	
Allergic diseases	
Asthma	14,087 (2.24)
AR	78,743 (12.5)
AD	10,962 (1.74)
Urbanization levels	
1 (highest)	191,780 (30.5)
2	201,557 (32.1)
3	114,312 (18.2)
4 (lowest)	121,229 (19.3)
Parity	
1	427,514 (68.0)
2	176,644 (28.1)
3+	24,720 (3.93)
Gestational age (week)	38.4 (1.52)
Birth weight, grams	3110 (427)
Mode of delivery	
Spontaneous birth	411,925 (65.5)
Cesarean section	216,953 (34.5)
Age at delivery (years)	29.1 (4.59)
Gestational diabetes	10,196 (1.62)
Preeclampsia	4663 (0.74)

*AR* allergic rhinitis, *AD* atopic dermatitis

### Maternal and perinatal impacts on childhood asthma

In multivariate logistical regression, we found that male gender (aOR 1.50, 95% CI 1.47–1.53), maternal asthma (aOR 1.80, 95% CI 1.71–1.89) and maternal AR (aOR 1.33, 95% CI 1.30–1.37) were independent risk factor for childhood asthma, and maternal AD (aOR 1.06, 95% CI 0.99–1.14) also tended to be associated with childhood asthma. A high urbanization level was positively associated with childhood asthma in a dose–response manner, whereas a high number of older siblings was inversely associated with childhood asthma. With regards to the perinatal factors, preterm birth (aOR 1.32, 95% CI 1.27–1.37), low birth weight (aOR 1.14, 95% CI 1.10–1.19) and cesarean section (aOR 1.10, 95% CI 1.08–1.13) were risk factors for childhood asthma, whereas a high maternal age at delivery appeared to be a protective factor (aOR 0.91, 95% CI 0.88–0.94). Taken together, these data provide evidence with regards to maternal and perinatal impacts on childhood asthma, including male gender, maternal asthma, maternal AR, a high urbanization level, a low number of older siblings, preterm birth, low birth weight, and cesarean section (Table 2).

**Table 2:** Crude and adjusted odds ratios for the association between variables and the risk of childhood asthma

	Children without asthma n = 584,963	Children with asthma n = 43,915	Crude OR (95% CI)	Adjusted OR (95% CI)	p value
Child sex					
Female	283,358 (48.4)	16,932 (38.6)	Ref	Ref	–
Male	301,605 (51.6)	26,983 (61.4)	1.50 (1.47–1.53)	1.50 (1.47–1.53)	< 0.0001
Maternal allergic diseases					
Asthma	12,231 (2.09)	1856 (4.23)	2.07 (1.97–2.17)	1.80 (1.71–1.89)	< 0.0001
AR	71,465 (12.2)	7278 (16.6)	1.43 (1.39–1.47)	1.33 (1.30–1.37)	< 0.0001
AD	10,108 (1.73)	854 (1.94)	1.13 (1.05–1.21)	1.06 (0.99–1.14)	0.09
Urbanization levels					
1 (highest)	175,943 (30.1)	15,837 (36.1)	1.59 (1.54–1.64)	1.59 (1.54–1.64)	< 0.0001
2	186,731 (31.9)	14,826 (33.8)	1.40 (1.36–1.44)	1.40 (1.35–1.44)	< 0.0001
3	107,558 (18.4)	6754 (15.4)	1.11 (1.07–1.15)	1.11 (1.07–1.14)	< 0.0001
4 (lowest)	114,731 (19.6)	6498 (14.8)	Ref	Ref	–
No. of siblings					
0	396,932 (67.9)	30,582 (69.6)	Ref	Ref	–
1	164,789 (28.2)	11,855 (27.0)	0.93 (0.91–0.95)	0.94 (0.92–0.96)	< 0.0001
2 or more siblings	23,242 (3.97)	1478 (3.37)	0.83 (0.78–0.87)	0.85 (0.81–0.90)	< 0.0001
Preterm birth (< 37 weeks)					
No	545,976 (93.3)	39,843 (90.7)	Ref	Ref	–
Yes	38,987 (6.66)	4072 (9.27)	1.43 (1.38–1.48)	1.32 (1.27–1.37)	< 0.0001
Low birth weight (< 2500 g)					
No	547,739 (93.6)	40,435 (92.1)	Ref	Ref	–
Yes	37,224 (6.36)	3480 (7.92)	1.27 (1.22–1.31)	1.14 (1.10–1.19)	< 0.0001
Mode of delivery					
Spontaneous birth	384,343 (65.7)	27,582 (62.8)	Ref	Ref	
Cesarean section	200,620 (34.3)	16,333 (37.2)	1.13 (1.11–1.16)	1.10 (1.08–1.13)	< 0.001
Maternal age at delivery (years)					
< 35	522,943 (89.4)	39,363 (89.6)	Ref	Ref	–
≥ 35	62,020 (10.6)	4552 (10.4)	0.98 (0.94–1.01)	0.91 (0.88–0.94)	< 0.0001
Gestational diabetes	9500 (1.62)	696 (1.58)	0.98 (0.9–1.05)	0.94 (0.87–1.01)	0.14
Preeclampsia	4259 (0.73)	404 (0.92)	1.27 (1.14–1.40)	1.06 (0.95–1.17)	0.17

*AR* allergic rhinitis, *AD* atopic dermatitis

### Shared prenatal impacts among childhood allergic diseases

Given that both maternal asthma and AR were independently associated with childhood asthma, we thus aimed to address the potential shared maternal impacts among childhood asthma, AR, and AD. We found that the association between maternal asthma and childhood asthma (aOR 1.80, 95% CI 1.71–1.89) was stronger compared with that between maternal asthma and childhood AR (aOR 1.67, 95% CI 1.50–1.87) as well as childhood AD (aOR 1.31, 95% CI 1.22–1.40). Similarly, the association between maternal AR and childhood AR (aOR 1.62, 95% CI 1.53–1.72) was higher than that between maternal AR and childhood asthma (aOR 1.33, 95% CI 1.30–1.37) as well as childhood AD (aOR 1.35, 95% CI 1.31–1.40). A similar pattern of associations was also found between maternal AD and childhood AD (aOR 1.62, 95% CI 1.51–1.75), asthma (aOR 1.06, 95% CI 0.99–1.14), and AR (aOR 1.37, 95% CI 1.18–1.58) (Table 3). In addition to the shared maternal impacts among childhood allergic diseases, we also found largely similar perinatal impacts among the three childhood allergic diseases (Additional file 1: Table S1). Furthermore, we also examined the relationships between the number of maternal allergic diseases and the three childhood allergic diseases. We found that the number of maternal allergic diseases was associated with the three childhood allergic diseases in a dose–response manner (Table 4). Collectively, these data demonstrated the shared maternal impacts among childhood allergic diseases, including asthma, AR, and AD.

**Table 3:** Associations between maternal allergic diseases and childhood allergic diseases

Maternal status	Childhood allergic diseases		
	Asthma	AR	AD
	OR (95% CI)	OR (95% CI)	OR (95% CI)
Asthma			
No	Ref	Ref	Ref
Yes	1.80 (1.71–1.89)	1.67 (1.50–1.87)	1.31 (1.22–1.40)
AR			
No	Ref	Ref	Ref
Yes	1.33 (1.30–1.37)	1.62 (1.53–1.72)	1.35 (1.31–1.40)
AD			
No	Ref	Ref	Ref
Yes	1.06 (0.99–1.14)	1.37 (1.18–1.58)	1.62 (1.51–1.75)

Model was adjusted for sex, urbanization levels, pregnant age, preterm birth, low birth weight, cesarean section, number of siblings, gestational diabetes, and preeclampsia

*AR* allergic rhinitis, *AD* atopic dermatitis

**Table 4:** Associations between the number of cumulative maternal allergic diseases and childhood allergic diseases

Maternal status	Childhood allergic diseases		
	Asthma	AR	AD
	OR (95% CI)	OR (95% CI)	OR (95% CI)
No. of allergic disease			
0	Ref	Ref	Ref
1	1.35 (1.31–1.38)	1.58 (1.50–1.68)	1.35 (1.31–1.40)
2	2.12 (2.00–2.25)	2.56 (2.27–2.89)	1.86 (1.72–2.01)
3	2.41 (1.81–3.19)	4.11 (2.53–6.69)	2.81 (2.03–3.89)

Model was adjusted for sex, urbanization levels, pregnant age, preterm birth, low birth weight, cesarean section, the number of siblings, gestational diabetes, and preeclampsia

*AR* allergic rhinitis, *AD* atopic dermatitis

## Discussion

In this population-based study, we investigated the maternal and perinatal impacts on childhood asthma and the potential shared impacts among the three prevalent childhood allergic diseases including asthma, AR, and AD. We found that male gender, maternal asthma, maternal AR, a high urbanization level, preterm birth, low birth weight and cesarean section were independently associated with the development of childhood asthma, whereas a high number of older siblings and a high maternal age at delivery were negatively associated with childhood asthma. Moreover, we delineated the shared maternal impacts among the three prevalent childhood allergic diseases in the present study.

The impact of maternal and perinatal factors on childhood allergic diseases can be attributed to both genetic susceptibility and gene–environment interactions [17]. Indeed, asthma, AR and AR are prevalent atopic disorders of complex etiology, and these three diseases are frequently observed as the atopic march, indicating the potential shared underlying immunological mechanisms among these three allergic diseases [7]. Ferreira et al. [8], conducting a large-scale GWAS (n = 360,838) study in which any one of asthma, AR and AD was considered as the presence of the allergic disease, identified 136 independent risk variants for allergic diseases. Of the 136 variants, 73 novel genetic variants were not found in the previous GWAS research of individual allergic diseases, and the novel approach and findings of Ferreira et al. [8] highlight the existence of shared genetic risk variants that can be involved in the expression of allergy-related genes in asthma, AR and AD. Similarly, Zhu et al. [9], analyzing a number of large-scale GWAS databases including the UK Biobank and GABRIEL consortium study, found a strong genome-wide genetic correlation between asthma and other allergic diseases including AR and AD (r_g_ = 0.75, P = 6.84 × 10^−62^) through using linkage disequilibrium score regression, and their findings further highlight the shared genetic architecture of asthma and other allergic diseases including AR and AD. Through using the two population-based databases, our findings that showed the shared maternal impacts among asthma, AR and AD further provide the epidemiological evidence and support the shared underlying biological pathways among these three allergic diseases.

Perinatal environmental factors and gene–environment interactions have been implicated in the development of childhood allergic diseases in addition to genetic factors [17, 18]. In line with our data, previous studies have shown that a high urbanization level was associated with childhood allergic diseases, so-called hygiene theory [19, 20]. Advances in genome sequencing technologies enable researchers to explore the commensal microbiota in the past decades, and increasing evidence have shown the crucial role of commensal microbiota in the pathogenesis of allergic diseases [21, 22]. Notably, Stokholm et al. [23] recently reported that 1-year-old children with an immature microbial composition in the gut had an increased risk of asthma at age 5 years; however, such association only existed among children born by mothers with asthma. This intriguing and crucial finding indicates that the maternal factor may affect the development of childhood asthma through both genetic predisposition and gene-environment interaction. Similarly, Ta et al. [24], investigating nasal microbiome, also found that maturation of the nasal microbiome in the first 18 months of life was associated with the development of childhood rhinitis and concomitant wheeze. Therefore, the association between maternal and childhood allergic disease as we shown in this study might result from both genetic and environmental factors, particularly the commensal microbiota.

The sibling effect on asthma has been found in a number of global studies, including International Study of Asthma and Allergies in Childhood (ISAAC), GABRIEL Consortium (A Multidisciplinary Study to Identify the Genetic and Environmental Causes of Asthma in the European Community) and PASTURE (Protection against Allergy Study in Rural Environments), and we also observed an inverse association between number of older siblings and childhood asthma [25–27]. The hygiene hypothesis has been attributed to the sibling effect in asthma, and Laursen et al. [28], further identified that having older siblings was associated with gut microbiota development during early childhood. Therefore, we thought that environmental effects including microbiome-associated effects may also underlie the sibling effect in childhood asthma in addition to genetic effects.

Sex has been implicated with the development and severity of asthma in an age-dependent manner, with the pre-school boy tend to have a higher incidence of asthma than those in the pre-school girl, whereas an increasing prevalence and severity of asthma were found in girls after puberty [29, 30]. Given that we focused on childhood asthma in the present study, we hence found that male gender was an independent risk factor for incident asthma. Interestingly, we noted that a high maternal age at delivery was a protective factor for asthma in this study. Similar to our finding, one recently published large-scale study, investigating 10,692 adults from 13 European countries, reported that increasing maternal age at delivery was associated with increasing forced expiratory volume in 1 s (FEV_1_) and a low incidence of asthma at age 25–55 years, and the inverse correlation between maternal age at delivery and incident asthma mainly existed in females [31]. Additionally, we speculated that environmental factors as the aforementioned discussion regarding sibling effect on asthma might also at least partly explain the inverse association between maternal age at delivery and incident childhood asthma.

Notably, there is a wide range of reported prevalence of childhood asthma among studies due to the varied definitions for childhood asthma/wheezing, and such a wide range of the reported prevalence reflects the difficult to accurately diagnose asthma in children and the complex of childhood wheezing [32]. In studies using claim data, the reported prevalence was nearly 20% using criteria with at least two out ambulatory visits or one hospitalization for asthma [33], while approximately 10% using the strict criteria with diagnosis of asthma after 5 years of age [34] or merely 6% after restricted by the ever prescription of ICS, which is the fundamental therapy in asthma and mainly prescribed by the pulmonologist/allergist [12, 35]. In this study, we used stringent criteria with the ever prescription of ICS, and the prevalence of asthma (6.98% till age 6) in this population-based study should be accurate.

This study has limitations. First, the diagnosis of asthma was physician-diagnosed asthma without confirmation by a pulmonary function; however, the diagnosis of asthma should be accurate given that all of asthmatics had received ICS in this study. Second, smoking history, a known risk perinatal factor for asthma [36], could not be assessed in the claim databases of this study, but we thought that smoking should not affect the trend of correlations identified in this study. Third, the impact of the maternal AD on childhood allergic diseases could not be delineated given that the prevalence of adulthood AD (1.74%) was relatively low.

In conclusion, this population-based study provides robust epidemiological evidence regarding the prenatal risk factors for childhood asthma, namely male gender, maternal asthma, maternal AR, a high urbanization level, a low number of siblings, preterm birth, low birth weight, and cesarean section. Additionally, we also demonstrated the shared maternal impacts among childhood allergic diseases, including asthma, AR, and AD, and these data support the need to survey coexisting allergic diseases, particularly among children whose mother also has allergic diseases. These findings shed lights on the prenatal impacts on the development of childhood allergic diseases, and more studies are warranted to investigate the pivotal immunological pathway and the complex gene-environment interaction in the pathogenesis of childhood allergic diseases.

## Supplementary information

**Additional file 1: Table S1.** Associations between perinatal variables and childhood allergic diseases.

## Data Availability

All data analyzed in this study are included in the manuscript.

## Abbreviations

AD: atopic dermatitis
aOR: adjusted odds ratio
AR: allergic rhinitis
CI: confidence interval
FEV1: forced expiratory volume in 1 s
GWAS: genome-wide association studies
ICD-9-CM: International Classification of Diseases, 9th Revision, Clinical Modification (ICD-9-CM)
ICS: inhaled corticosteroid
ISAAC: International Study of Asthma and Allergies in Childhood
NHI: National Health Insurance
NHIRD: National Health Insurance Research Database
NHRI: National Health Research Institutes
PASTURE: Protection against Allergy Study in Rural Environments
SD: standard deviation
TBCS: Taiwan Birth Cohort Study
