# Supplementary material for: Shared prenatal impacts among childhood asthma, allergic rhinitis and atopic dermatitis: a population-based study
Source: Allergy Asthma Clin Immunol. 2019 Sep 3;15:52. doi: 10.1186/s13223-019-0365-y (PMC6724237; doi:10.1186/s13223-019-0365-y)
Supplement: Supplementary file 1 — Additional file 1: Table S1. Associations between perinatal variables and childhood allergic diseases. [file 13223_2019_365_MOESM1_ESM.docx]

| **Table S1. Associations between perinatal variables and childhood allergic diseases** | | | | | | |
| --- | --- | --- | --- | --- | --- | --- |
|  |  | **Childhood allergic diseases** | | | | |
|  |  | **Asthma** |  | **AD** |  | **AR** |
| **Perinatal factors** |  | **OR (95% CI)** |  | **OR (95% CI)** |  | **OR (95% CI)** |
| Urbanization levels |  |  |  |  |  |  |
| 1 (highest) |  | 1.59 (1.54-1.64) |  | 1.74 (1.67-1.80) |  | 1.07 (1.00-1.14)^a^ |
| 2 |  | 1.40 (1.35-1.44) |  | 1.34 (1.28-1.39) |  | 1.09 (1.02-1.17) |
| 3 |  | 1.11 (1.07-1.14) |  | 1.14 (1.09-1.20) |  | 1.01 (0.94-1.09) |
| 4+ (lowest) |  | ref |  | ref |  | ref |
| No. of siblings |  |  |  |  |  |  |
| 0 |  | ref |  | ref |  | ref |
| 1 |  | 0.94 (0.92-0.96) |  | 0.75 (0.73-0.77) |  | 0.66 (0.63-0.70) |
| Over 2 |  | 0.85 (0.81-0.90) |  | 0.59 (0.55-0.64) |  | 0.66 (0.57-0.75) |
| Preterm birth (<37 weeks) |  |  |  |  |  |  |
| No |  | ref |  | ref |  | ref |
| Yes |  | 1.32 (1.27-1.37) |  | 0.99 (0.94-1.05) |  | 1.07 (0.98-1.18) |
| Low birth weight (<2500g) |  |  |  |  |  |  |
| No |  | ref |  | ref |  | ref |
| Yes |  | 1.14 (1.10-1.19) |  | 0.93 (0.88-0.99) |  | 1.12 (1.01-1.23) |
| Model of delivery |  |  |  |  |  |  |
| Spontaneous birth |  | ref |  | ref |  | ref |
| Cesarean section |  | 1.10 (1.08-1.13) |  | 1.02 (1.00-1.05)^b^ |  | 1.12 (1.06-1.17) |
| Maternal age at delivery |  |  |  |  |  |  |
| <35 |  | ref |  | ref |  | ref |
| ≥35 |  | 0.91 (0.88-0.94) |  | 1.11 (1.07-1.15) |  | 1.13 (1.06-1.22) |
| Gestational diabetes |  |  |  |  |  |  |
| No |  | ref |  | ref |  | ref |
| Yes |  | 0.94 (0.87-1.01) |  | 1.11 (1.01-1.22) |  | 1.12 (0.95-1.32) |
| Preeclampsia |  |  |  |  |  |  |
| No |  | ref |  | ref |  | ref |
| Yes |  | 1.06 (0.95-1.17) |  | 1.00 (0.87-1.15) |  | 0.76 (0.58-1.01) |

^a^p = 0.06; ^b^p = 0.11. Abbreviations: AR, allergic rhinitis; AD, atopic dermatitis
